# Comparative Efficacy and Long-Term Outcomes of Drug-Eluting Stents vs. Bare-Metal Stents in Coronary Artery Disease: A Systematic Review

**DOI:** 10.7759/cureus.86617

**Published:** 2025-06-23

**Authors:** Hina Amjad, Asad Ullah, Mehtab Shakoor, Faiqa Tanveer, Mehak Qureshi, Fazeelat Jahan, Hamna Manzoor, Mohamed H Mahmoud, Fathima Masharifa Ahamed, Anik Halder, Rimsha Dilawar, Nouman Anthony

**Affiliations:** 1 Internal Medicine, Nazir Bhatti Memorial Hospital, Abbottabad, PAK; 2 Cardiology, Belfast Health and Social Care Trust, Belfast, GBR; 3 Medicine and Surgery, Liaquat National Hospital and Medical College, Karachi, PAK; 4 Medicine and Surgery, University College of Medicine and Dentistry, Lahore, PAK; 5 Surgery, Government Medical College, Srinagar, Srinagar, IND; 6 Obstetrics and Gynaecology, Shadan Institute of Medical Sciences, Hyderabad, IND; 7 General Medicine, Rehman Medical Institute, Peshawar, PAK; 8 General Surgery, El-Salam Specialized Hospital, Cairo, EGY; 9 Internal Medicine, Ras Al Khaimah Medical and Health Sciences University, Abu Dhabi, ARE; 10 Internal Medicine, Dhaka Medical College Hospital, Dhaka, BGD; 11 Community Medicine, Fauji Foundation Hospital, Rawalpindi, PAK; 12 General Medicine, Rehman Medical Institue, Peshawar, PAK

**Keywords:** bare-metal stents, coronary artery disease, drug-eluting stents, dual antiplatelet therapy, major adverse cardiovascular events, myocardial infarction, percutaneous coronary intervention, restenosis, stent thrombosis, target lesion revascularization

## Abstract

Coronary artery disease (CAD) is a major global cause of morbidity and mortality, driving ongoing improvements in percutaneous coronary intervention (PCI). Drug-eluting stents (DES) have largely replaced bare-metal stents (BMS) due to superior performance in reducing restenosis and the need for repeat revascularization. This systematic review compares the efficacy and long-term safety of DES versus BMS in patients undergoing PCI. A total of 13 studies, including randomized controlled trials and observational studies, were included, covering diverse populations and follow-up periods ranging from 1 to 14 years.

Primary outcomes assessed were all-cause mortality, major adverse cardiovascular events (MACE), myocardial infarction (MI), and target lesion revascularization (TLR). DES, particularly second-generation devices, were consistently associated with lower rates of MACE, MI, and TLR compared to BMS. Everolimus-eluting stents (EES) showed the most favorable safety profile regarding stent thrombosis. Secondary outcomes included in-stent restenosis (ISR) and bleeding risks. Notably, the benefits of DES were more pronounced in patients with diabetes and complex coronary anatomy. Some studies suggested gender-based differences favoring DES in women, though subgroup findings were exploratory and not always powered for statistical comparison. A few long-term studies observed a narrowing of the efficacy gap between DES and BMS beyond 10 years, though the clinical relevance of this remains limited.

Overall, the findings support the continued use of DES as the preferred option in most PCI settings, with individualized decisions based on lesion complexity, comorbidities, and patient adherence to dual antiplatelet therapy. Continued innovation in stent design and long-term patient monitoring remains essential to optimizing CAD outcomes.

## Introduction and background

Coronary artery disease (CAD) remains a leading cause of morbidity and mortality worldwide [1], accounting for approximately 610,000 deaths annually [2]. The primary etiology, atherosclerosis, causes progressive narrowing and obstruction of coronary arteries, leading to myocardial ischemia, infarction, and heart failure. Percutaneous coronary intervention (PCI) has become a cornerstone in CAD management, with coronary stents playing a pivotal role in restoring perfusion and alleviating symptoms [3]. Technological advancements in stent design have shifted clinical practice from bare-metal stents (BMS) to drug-eluting stents (DES), driven by their demonstrated ability to reduce restenosis and the need for repeat revascularization [4,5].

DES are coated with antiproliferative drugs that inhibit neointimal hyperplasia, the primary mechanism underlying restenosis. As a result, DES has largely replaced BMS in routine clinical use across most developed healthcare systems [6]. However, their long-term superiority has come under scrutiny due to persistent concerns about late stent thrombosis, the need for prolonged dual antiplatelet therapy (DAPT), bleeding risk, and cost-effectiveness, particularly in resource-limited settings [7]. These concerns are particularly relevant in patients with contraindications to long-term DAPT, those at high bleeding risk, or when treating large-caliber vessels where restenosis risk is inherently low. In such scenarios, BMS may still be considered, although this remains a topic of ongoing debate.

Despite the clear efficacy of second-generation DES, uncertainties remain regarding their performance over extended durations-commonly defined as follow-up periods exceeding five years. Some studies suggest that the clinical outcome gap between DES and BMS narrows beyond this timeframe, especially in low-risk anatomical and clinical contexts. However, this phenomenon lacks robust supporting evidence and requires further validation. Moreover, there are clinical situations where DES may not offer a distinct advantage-such as in large, non-complex lesions or in patients unable to adhere to long-term antiplatelet regimens-where BMS may remain a viable alternative. Clarifying these contexts is central to optimizing patient-specific stent selection.

An additional gap in current literature concerns the underrepresentation of sex-based outcome data. Although some trials have explored stent performance in women, gender-specific subgroup analyses are often underpowered or not pre-specified. This limits the ability to draw definitive conclusions about potential differences in efficacy or safety between men and women following stent placement [8]. A deeper understanding of how patient sex may influence stent outcomes could inform more personalized treatment strategies.

Another area requiring clarification is the use of DES in treating in-stent restenosis (ISR). The term “DES-ISR” typically refers to restenosis occurring within previously placed DES, which presents distinct clinical challenges compared to de novo lesions. In contrast, BMS have limited effectiveness in ISR scenarios, and DES or drug-eluting balloons are generally preferred in this setting [9]. Clear distinctions between ISR treatment and initial stent selection need to be maintained when comparing these technologies.

Given the evolving landscape of interventional cardiology, this systematic literature review (SLR) aims to provide an updated comparison of DES versus BMS in terms of major adverse cardiovascular events (MACE), all-cause mortality, myocardial infarction (MI), and target lesion revascularization (TLR) [10]. It also evaluates the risks of stent thrombosis and examines patient subgroup outcomes based on factors such as age, sex, and comorbidities. Importantly, this review explores specific clinical contexts where BMS may remain relevant and examines the durability of DES benefits over long-term follow-up. By synthesizing current evidence, this review aims to bridge existing knowledge gaps, guide patient-centered decision-making, and support future advancements in stent technology for CAD management.

## Review

Materials and methods

Search Strategy

A comprehensive search strategy was employed to identify relevant studies comparing DES and BMS in CAD. Major electronic databases, including PubMed, EBSCO, Cochrane Library, and Google Scholar, were systematically searched for peer-reviewed articles published between January 2019 and October 2024. This date range was selected to ensure inclusion of the most recent and clinically relevant evidence, particularly studies evaluating second-generation DES and long-term outcomes beyond earlier trials. A combination of Medical Subject Headings (MeSH) terms and Boolean operators was used to optimize search results, incorporating keywords such as “drug-eluting stents” OR “DES,” “bare-metal stents” OR “BMS,” “percutaneous coronary intervention” OR “PCI,” “coronary artery disease” OR “CAD,” “myocardial infarction,” “stent thrombosis,” and “revascularization.” Study selection followed PRISMA guidelines, ensuring a rigorous screening process based on predefined inclusion and exclusion criteria, with final selections including randomized controlled trials (RCTs), cohort studies, and meta-analyses that reported on key clinical outcomes such as mortality, MACE, MI, TLR, and stent thrombosis.

Study Design

This systematic literature review includes both RCTs and non-randomized study interventions (NRSIs). RCTs, regarded as the gold standard for clinical research, enhance the validity of findings by minimizing bias and ensuring robust comparisons. Meanwhile, NRSIs, including cohort studies, meta-analyses, and retrospective studies, contribute valuable real-world evidence, capturing a broader spectrum of clinical scenarios and patient demographics. This mixed-methods approach enables a balanced assessment of both controlled experimental data and observational insights, strengthening the applicability of the findings.

The study follows the Population, Intervention, Comparison, Outcome, and Time (PICOT) framework to define the research scope. The population includes adults (≥18 years) diagnosed with CAD undergoing percutaneous coronary intervention (PCI) with stent implantation. The intervention focuses on first- and second-generation DES, while the comparator group consists of patients receiving BMS. Key clinical outcomes assessed include efficacy metrics such as mortality, MI, TLR, and MACE, alongside safety considerations including stent thrombosis, bleeding risk, and other adverse events. Additionally, subgroup analyses are conducted to evaluate the impact of stent choice in specific populations, such as elderly patients and gender-based differences. The follow-up duration across the included studies ranges from 1 to 14 years, allowing for both short-term and long-term comparisons of stent performance. By integrating RCTs and NRSIs within a structured PICOT framework, this study ensures a rigorous and multifaceted approach, addressing both controlled trial findings and broader real-world outcomes to provide clinically relevant conclusions for CAD management.

Eligibility Criteria

The eligibility criteria for this systematic literature review were defined to ensure the inclusion of high-quality, relevant studies comparing DES and BMS in the management of CAD. Only peer-reviewed RCTs, cohort studies, and meta-analyses published between January 2019 and September 2024 were considered. Studies had to be published in English and directly compare DES and BMS in adult patients (≥18 years) undergoing PCIs. To maintain methodological rigor, included studies were required to report at least one of the key clinical outcomes, such as mortality, MI, TLR, ST, MACE, or bleeding risks. Additionally, a minimum follow-up duration of 12 months was mandated to ensure sufficient observation of clinical outcomes.

Studies were excluded if they were not published in English, conducted before 2019, or did not directly compare DES and BMS. Non-peer-reviewed publications, including conference abstracts, editorials, letters, and case reports, were also excluded. Furthermore, studies exclusively evaluating other stent types, such as bioresorbable stents, or those lacking sufficient quantitative data or detailed outcome reporting, were omitted. Research involving non-human subjects or in vitro models was also excluded, as they do not provide clinically applicable insights into PCI outcomes. These strict inclusion and exclusion criteria ensure a focused and high-quality evidence base, allowing for a robust comparison of DES and BMS in real-world and controlled clinical settings.

Quality Assessment

The quality of the included studies was assessed using the Cochrane Risk of Bias (RoB) tool for randomized controlled trials (RCTs) and the Newcastle-Ottawa Scale (NOS) for cohort studies. These tools evaluated factors such as selection bias, performance bias, detection bias, and attrition bias to ensure methodological rigor. The majority of studies demonstrated a low risk of bias across most domains, indicating a high level of reliability and validity in the findings [11-15].

Data Analysis

A hybrid analytical approach was employed, combining narrative synthesis with quantitative findings [14]. This method accounted for heterogeneity across study designs, follow-up durations, and patient populations, ensuring a comprehensive evaluation of clinical outcomes. Additionally, subgroup analyses were conducted to explore variations in efficacy and safety among different patient demographics, such as age, gender, and comorbid conditions.

Data Extraction and Synthesis

Data extraction and synthesis were conducted systematically. A structured table was used to capture key variables, including study design, sample size, clinical outcomes, primary findings, effect sizes, and follow-up durations. The extracted data were synthesized both qualitatively and quantitatively, with effect sizes compared across studies to identify trends and variations in outcomes. However, due to heterogeneity in study designs, follow-up periods, and patient populations, meta-analytic techniques were not applied. Instead, a narrative synthesis approach was utilized to integrate findings and provide meaningful insights into the comparative effectiveness and safety of DES and BMS.

Results

Study Selection Process

**Figure 1:**
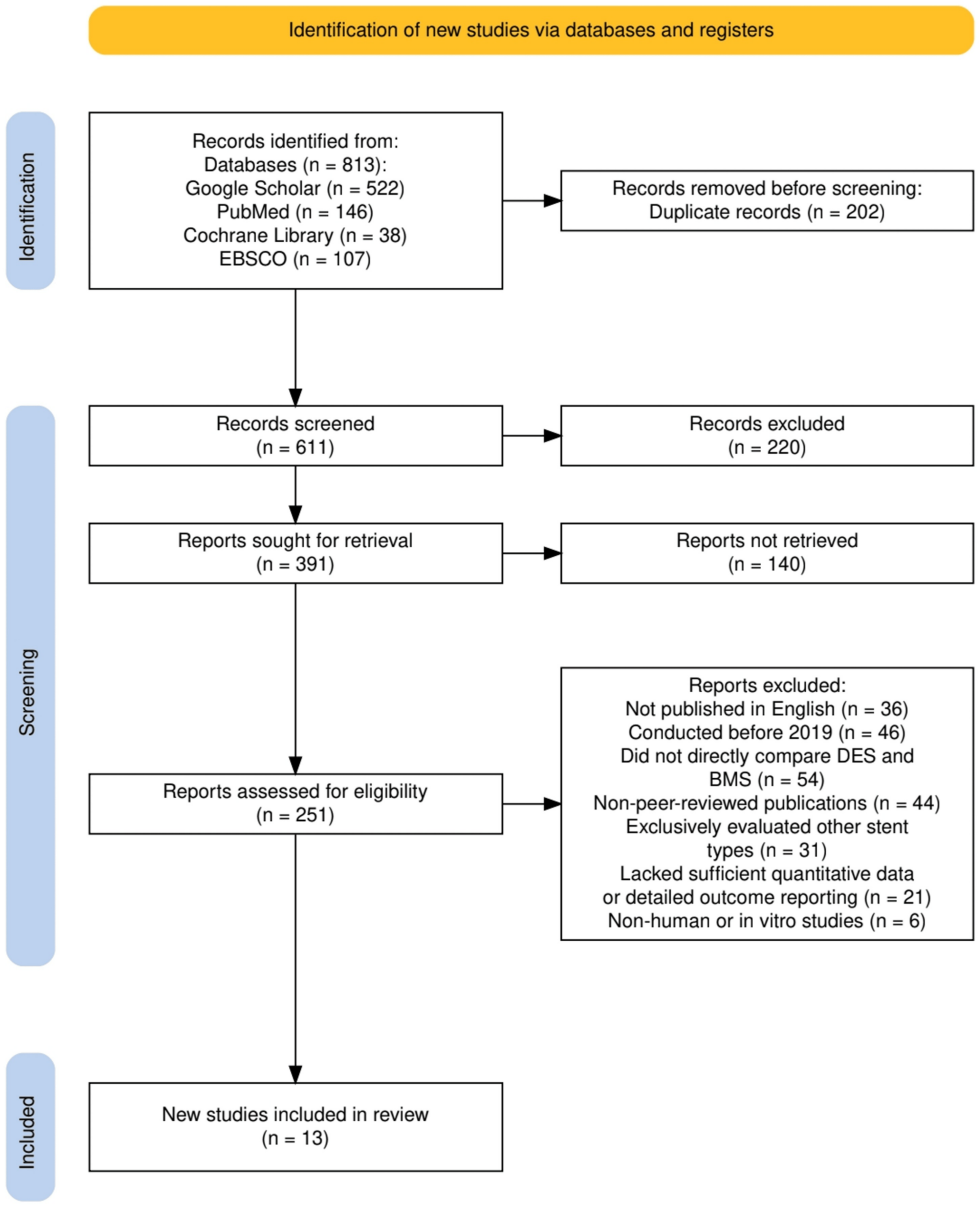
The PRISMA flowchart represents the study selection process.

The study selection process is detailed in Figure 1, which outlines the PRISMA 2020 flow diagram used in this review. A total of 813 records were identified through four electronic databases: Google Scholar (n=522), PubMed (n=146), Cochrane Library (n=38), and EBSCO (n=107). After removing 202 duplicate records, 611 records remained for screening based on titles and abstracts. Of these, 220 records were excluded for not meeting the initial inclusion criteria. The full texts of 391 reports were sought for retrieval, but 140 could not be accessed, leaving 251 reports for detailed eligibility assessment. Upon full-text evaluation, 238 reports were excluded for reasons including not being published in English (n=36), being conducted before 2019 (n=46), not directly comparing drug-eluting stents (DES) and bare-metal stents (BMS) (n=54), being non-peer-reviewed (n=44), evaluating other stent types (n=31), lacking sufficient quantitative data (n=21), or involving non-human or in vitro studies n=(6). Ultimately, 13 studies met all eligibility criteria and were included in the final systematic review.

Characteristics of the Selected Studies

The selected studies, summarized in Table 1, include RCTs, meta-analyses, cohort studies, and observational analyses, with sample sizes ranging from a few hundred to over 14,000 participants. The primary outcomes assessed include mortality, MACE, MI, TLR, and stent thrombosis, providing a comprehensive comparison of DES and BMS in CAD. Findings indicate that second-generation DES significantly reduce TLR, restenosis, and stent thrombosis rates compared to BMS, with some studies demonstrating sustained benefits over follow-up periods of up to 14 years. While the impact on all-cause mortality remains variable, subgroup analyses suggest that DES offer greater advantages in high-risk populations, such as elderly patients and those with diabetes. Certain studies also highlight gender-specific differences in stent efficacy, reinforcing the need for personalized treatment strategies. Notably, everolimus-eluting stents (EES) emerge as a superior option, offering enhanced safety and long-term efficacy. Despite these advancements, the narrowing efficacy gap between DES and BMS in some studies underscores the need for continued surveillance and patient-specific stent selection based on clinical and anatomical factors.

**Table 1:** The characteristics of the selected studies in the article. BMS, bare-metal stents; CAD, coronary artery disease; CD, cardiac death; DES, drug-eluting stents; DOCE, device-oriented composite endpoint; EES, everolimus-eluting stent; HR, hazard ratio; ID-TLR, ischemia-driven target lesion revascularization; ISR, in-stent restenosis; LAD/LM, left anterior descending / left main arteries; MACCE, major adverse cardiovascular and cerebrovascular events; MACE, major adverse cardiovascular events; MI, myocardial infarction; OR, odds ratio; P, probability value; POCE, patient-oriented composite endpoint; RCT, randomized controlled trial; RE-ISR, recurrent in-stent restenosis; RR, relative risk; ST, stent thrombosis; TLR, target lesion revascularization; TVR, target vessel revascularization; ZES, zotarolimus-eluting stent

Authors/Year	Study Design	Sample Size (DES/BMS)	Outcomes Measured	Primary Findings	Effect Sizes	Follow-Up Period
Changal et al., [16] (2021)	RCT, meta-analysis	6006 / 4291	All-cause mortality, MI, TLR, ST	Second-gen DES significantly reduced mortality (2.4% vs. 3.9%), TLR (3.5% vs. 8.6%), and MI (2.1% vs. 2.9%) compared to BMS.	OR for mortality: 0.74; OR for TLR: 0.38; OR for MI: 0.73	1 to 5 years
Piccolo et al., [17] (2019)	Individual patient data meta-analysis, RCTs	14,070 / 12,546	Cardiac death, MI, ST, TLR, all-cause mortality	DES reduced risk of MI (HR 0.79), cardiac mortality (HR 0.89), ST (HR 0.63), and TLR (HR 0.55) compared to BMS.	HR for primary outcome: 0.84; MI: HR 0.79; ST: HR 0.63; TLR: HR 0.55	Mean 3.2 years
Bjerking et al., [18] (2019)	Pooled analysis of 2 RCTs	3079 / 1526	Cardiac death, non-fatal MI, clinically driven TLR	DES showed greater MACE reduction in women (6.1% vs. 14.7%) than men (7.7% vs. 12.1%).	MACE reduction: women HR 0.36, men HR 0.62; TLR reduction: women HR 0.24, men HR 0.48	Median 2 years
Lafont et al., [19] (2020)	RCT (SENIOR trial)	596 / 604	All-cause mortality, MI, stroke, ID-TLR, bleeding	DES significantly lowered revascularization rates (2% vs. 7%) without increasing bleeding or thrombosis risk.	ID-TLR RR 0.35; Composite endpoint RR 0.90	Median 2 years
Kufner et al., [20] (2020)	RCT (ISAR-TEST-5 trial)	2002 / 1000	DOCE, POCE, ST	No significant differences in DOCE or POCE between polymer-free and durable polymer DES.	DOCE HR 1.01; POCE HR 0.94; ST HR 0.85	Median 10 years
Fahrni et al., [21] (2020)	Multinational RCT	89 / 84	MACE, CD, MI, TVR	Lower MACE rates at 1 and 5 years for DES vs. BMS, with fewer revascularization events in DES group.	1-year MACE HR 0.14; 5-year MACE HR 0.40; 1-5 year TVR HR 0.33	5 years
Singhal et al., [22] (2020)	Prospective cohort study	118 / 122	TLR/TVR, all-cause death, need for medical management, asymptomatic patients	No significant difference in clinical outcomes between DES and BMS in large coronary arteries.	TLR/TVR: p = 0.6492; All-cause death: p = 0.7098	1 year
Piccolo et al., [23] (2021)	Individual patient data meta-analysis	13,650 / 12,373	Composite of CD or myocardial infarction, all-cause mortality	New-generation DES reduced risk of CD and MI in LAD/LM arteries significantly.	LAD/LM: CD or MI, HR 0.76; No-LAD/LM: HR 0.93	6 years
Walse et al., [24] (2023)	Retrospective cohort study	100 / 100	MACCE, ST	MACCE was significantly lower in DES than in BMS at 1 year, but similar at 5, 10, and 14 years.	MACCE at 1 year: DES 3 vs. BMS 10 (P = 0.04); 14 years: DES 37 vs. BMS 36 (P = 0.88)	14 years
Yang et al., [25] (2020)	Systematic review and meta-analysis	6256 participants	TLR, TVR,CD, ST/RE-ISR, MACE, all-cause death, MI	DES-ISR was associated with higher TLR, TVR, CD, and MACEs compared to BMS-ISR.	TLR and TVR: P < 0.00001; CD: P = 0.02; ST/RE-ISR and MACEs: P < 0.00001	Long-term
Sambola et al., [26] (2020)	Systematic review and meta-analysis	10,353 patients	Major bleeding, MACE, TVR risk, antithrombotic therapy effects	DES did not significantly increase major bleeding risk but showed lower TVR risk than BMS.	Major bleeding: RR 1.07; MACE: RR 0.96; TVR: RR 0.78	Short- and long-term
Elsayed et al., [27] (2020)	Comparative cohort study	210 patients	MACE, MI, TLR, ST	EES had the lowest MACE rate (19%) compared to ZES (28.2%) and BMS (41.7%).	MACE rate: BMS 41.7%, ZES 28.2%, EES 19% (P=0.002); ST rate: BMS 7.5%, ZES 2.2%, EES 1.3% (P=0.004)	2 years
Park et al., [28] (2021)	Observational cohort study	1102 patients	Composite outcome (all-cause death or MI), mortality, MI, TLR	DES showed significantly lower death, MI, and TLR rates compared to BMS over 10 years.	Composite outcome: DES 27.9%, BMS 37.0% (HR 0.71, P=0.02); mortality: DES 20.6%, BMS 29.6% (HR 0.65, P=0.01)	10 years

Discussion

Mortality and Major Adverse Cardiovascular Events (MACE)

Second-generation DES have demonstrated significant reductions in mortality and MACE compared to BMS, as reported by Changal et al. [16] and further supported by Fahrni et al. [21]. These benefits likely reflect advancements in stent design, biocompatibility, and antiproliferative drug release. Ultrathin-strut DES have been shown to further enhance MACE reduction, especially among elderly and diabetic populations [29]. However, Piccolo et al. [17] noted that while DES improved MI and stent thrombosis outcomes, they did not significantly impact cardiac mortality, suggesting that long-term survival is influenced by broader clinical factors beyond stent choice.

A crucial modifier of DES performance is the duration of dual antiplatelet therapy (DAPT). As outlined by Sambola et al. [26], extended DAPT in high-risk patients is associated with reduced ischemic complications. El Abdallaoui et al. [30] reported that tailoring DAPT duration to patient risk profiles can reduce MACE by 24%, reinforcing the need for individualized antiplatelet strategies to maximize benefit while minimizing bleeding risk.

Target Lesion Revascularization (TLR) and Restenosis Prevention

Across multiple studies, including Lafont et al. [19] and Sambola et al. [26], DES were consistently superior to BMS in reducing TLR, due to improved drug-elution mechanisms and biocompatible polymer coatings. Kandzari et al. [31] supported this by demonstrating that bioresorbable polymer DES further lowered TLR rates by 2.6% compared to permanent polymer DES, likely owing to enhanced endothelial healing.

Nonetheless, restenosis persists in anatomically challenging cases. Yang et al. [25] observed that complex bifurcation lesions and diffuse disease remain susceptible to restenosis despite DES implantation. Adjunctive strategies such as drug-eluting balloons and hybrid approaches may be necessary in these high-risk scenarios, emphasizing that DES, while highly effective, do not entirely obviate the risk of repeat interventions in complex CAD.

Stent Thrombosis and Adverse Events

Reduced stent thrombosis remains a defining strength of newer-generation DES. Elsayed et al. [27] and Fahrni et al. [21] reported significantly lower thrombosis rates with DES, particularly with EES. Kufner et al. [32] identified a 54% reduction in thrombosis with biodegradable polymer EES compared to older models. However, thrombosis risk is not entirely mitigated. Prati et al. [33] and Malik et al. [34] highlighted the influence of DAPT non-compliance, suboptimal implantation, and complex anatomy in increasing thrombosis risk.

Importantly, Yang et al. [25] found higher adverse event rates in patients with ISR involving DES, underscoring the need for vigilant long-term monitoring and careful case selection. Walse et al. [24] further noted that differences in thrombosis outcomes between DES and BMS diminish beyond 14 years, potentially due to survivor bias and evolving post-procedural medical therapies. These findings emphasize the role of structured follow-up and strict adherence to antiplatelet therapy, particularly during the critical first year post-implantation.

Long-Term Durability and Follow-Up Strategies

Evidence from Park et al. [28] supports the sustained long-term efficacy of DES, showing reduced mortality, MI, and TLR over a 10-year follow-up. Yin et al. [35] similarly reported durable benefits at nine years. However, Walse et al. [24] observed that beyond 14 years, clinical outcomes between DES and BMS begin to converge. While intriguing, this observation should be interpreted cautiously, as it may reflect confounding variables such as late events, differential survival, and improvements in secondary prevention measures.

These findings underscore the importance of long-term surveillance strategies and ongoing patient engagement. As emphasized by Sambola et al. [26], long-term success with DES is heavily dependent on consistent follow-up and medication adherence. The evolving nature of coronary pathophysiology over time also suggests that durable benefits of stenting must be considered in the context of lifelong cardiovascular risk management.

Subgroup Considerations and Personalized Stent Selection

The role of personalized medicine in PCI is becoming increasingly relevant. Bjerking et al. [18] and Singhal et al. [22] observed gender-specific variations in stent efficacy, with women showing a greater reduction in MACE with DES than men. However, these findings were derived from subgroup analyses that were often post hoc and may lack statistical power. As such, they should be interpreted with caution and validated in future sex-stratified trials. Pérez-López et al. [36] further postulated that hormonal and anatomical differences could modulate DES performance, though these hypotheses remain exploratory.

In diabetic patients and those with complex coronary lesions, DES consistently demonstrate superior outcomes, making them the preferred option in these subgroups. Conversely, Singhal et al. [22] noted that in large-diameter coronary arteries (≥3.5 mm), BMS may provide comparable outcomes, particularly in low-risk patients or those with contraindications to prolonged DAPT. Although BMS are now largely obsolete in guideline-based practice, they may still have a niche role in select populations, such as patients at high bleeding risk or those unable to maintain long-term DAPT adherence.

These subgroup-specific findings support the integration of clinical, anatomical, and patient-centered factors into stent selection. A tailored approach that accounts for comorbidities, vessel size, DAPT tolerance, and sex-based physiological considerations is essential for optimizing PCI outcomes.

Limitations

This review is not without limitations. The included studies demonstrated variability in sample sizes, follow-up durations (ranging from 1 to 14 years), and endpoint definitions (e.g., MACE, TLR), introducing heterogeneity that limited quantitative synthesis. Additionally, studies employed differing effect measures (e.g., ORs, HRs, RRs), which were reported as presented but not statistically pooled due to methodological inconsistencies. While all studies met minimum quality thresholds using Cochrane RoB and Newcastle-Ottawa Scale tools, the strength of conclusions is influenced by the variability in study design and reporting quality.

Another limitation is the underrepresentation of certain subgroups--particularly elderly patients, women, and those with recurrent ISR--which restricts the generalizability of subgroup findings. Furthermore, the reliance on narrative synthesis may reduce interpretative precision, though it was necessary due to the heterogeneity of the dataset.

Clinical Implications and Future Directions

This systematic review reinforces that DES remain the standard of care for most PCI procedures, particularly among patients with high-risk features such as diabetes, small vessel disease, or complex anatomy. EESs, in particular, offer favorable outcomes in terms of safety and efficacy. However, personalized stent selection--guided by lesion characteristics, comorbidity profiles, vessel size, and DAPT adherence--remains central to optimal outcomes.

Despite technological advancements, challenges persist in subgroups with ISR, complex bifurcation lesions, or contraindications to DAPT. These cases highlight the ongoing need for novel stent platforms, improved pharmacotherapy, and hybrid strategies. Future research should prioritize prospective, subgroup-focused trials and long-term ISR outcome studies to fill current evidence gaps.

## Conclusions

This systematic review confirms that second-generation DES offer superior outcomes compared to BMS in reducing TLR, MACE, and stent thrombosis in CAD patients. However, the long-term effectiveness of DES may vary across patient subgroups, emphasizing the importance of individualized stent selection based on lesion complexity, comorbidities, vessel size, and DAPT tolerance. Clinically, DES should remain the default strategy in most PCI cases, while BMS may still be considered in specific contexts, such as contraindications to prolonged antiplatelet therapy. Future research must prioritize large-scale, subgroup-specific trials, long-term studies on ISR, and the development of next-generation stent platforms and adjunctive pharmacotherapies. Translational efforts should focus on integrating precision medicine into interventional cardiology to enhance safety, durability, and personalized patient care.
